# The effect of different treatments of lymph after intestinal ischemia-reperfusion in rats on macrophages *in vitro*

**DOI:** 10.1371/journal.pone.0211195

**Published:** 2019-01-25

**Authors:** Rui Zhang, Guizhen He, Yukang Wang, Jie Wang, Wei Chen, Yingchun Xu

**Affiliations:** 1 Department of Parenteral and Enteral Nutrition, Peking Union Medical College Hospital, Chinese Academy of Medical Sciences and Peking Union Medical College (CAMS and PUMC), Beijing, China; 2 Department of Clinical Laboratory, Peking Union Medical College Hospital, Chinese Academy of Medical Sciences and Peking Union Medical College (CAMS and PUMC), Beijing, China; University of PECS Medical School, HUNGARY

## Abstract

**Background:**

To observe the effects of different treatments of lymph after intestinal I/R in rats on macrophages *in vitro*.

**Methods:**

Forty-eight healthy SPF SD rats weighing 300 ± 20 g, were randomly divided into two groups: group A, and group B. The rats in group A were drained of lymph fluid for 180 min; the rats in group B were subjected to 60 min ischemia by clamping the SMA, followed by 120 min reperfusion and 180 min of lymph drainage. The lymph fluid collected was divided into 4 sub-groups: 1. no treatment (A1, Ly, and B1, I/R Ly); 2. protein degradation (A2, Ly PD, and B2 I/R PD); 3. endotoxin removal (A3, Ly ER, and B3, I/R ER); 4. protein degradation plus endotoxin removal (A4, Ly PD+ER, and B4, I/R PD+ER), then used to stimulate a monocyte-macrophage cell line.

**Results:**

Compared with group A1, the levels of the inflammatory cytokines, chemokines, HMGB1 concentration, protein and mRNA expression of TLR4, HMGB1 and NF-κBp65 were significantly increased in group B1. There was a significant reduction in proinflammatory cytokines and of the expression of TLR4, NF-κBp65, and chemokines in groups A2, B2, A4, and B4. However, there were no significant decrease of these factors in groups A3 and B3.

**Conclusions:**

The lymph fluid drained after intestinal I/R can cause inflammation *in vivo* and *in vitro*. Deproteinization of lymph fluid with proteinase K significantly reduced the concentration of proinflammatory cytokines, chemokines, TLR4 and NF-κBp65 in cell culture supernatant, exerting a protective effect on inflammatory reaction caused by the intestinal I/R. Passage of lymph fluid through an endotoxin removal column did not reduce the levels of active proinflammatory factors produced by macrophages *in vitro*.

## Introduction

Ischemia-reperfusion (I/R) injury is a condition that causes cellular injury and ultimately tissue damage when the blood flow to the tissue is blocked and then resumed. Distant tissue injury, organ failure, or even deaths are observed after I/R [1]. A common event in most critical conditions is gut hypoperfusion with subsequent intestinal I/R injury to enterocytes and their supporting structures. The damage to and apoptosis of intestinal mucosal epithelial cells lead to the loss of basement membrane integrity and barrier function, which promote bacterial translocation and the local production of cytokines [2]. In recent years, numerous studies have validated that the transmission of proinflammatory mediators is through the intestinal lymphatics. Intestinal lymph fluid plays an important role in linking the visceral I/R injury and functional organ damage [3–5]. The “gut-lymph” pathway is therefore important in distant organ injury. However, the factors causing injury remain unknown [6]. Possible factors include Toll-like receptor 4 (TLR4), high-mobility group box 1 protein (HMGB1), endotoxin, as well as bioactive non-microorganism derived proteins and lipoproteins.

This research aimed to observe the effects of gut lymph fluid drained from rats after intestinal I/R on macrophages *in vitro*. Proinflammatory changes in both cells and culture supernatants were investigated. We could thus explore the mechanism of I/R-induced inflammation and identify the related proinflammatory bioactive factors.

## Materials and methods

### Animals and groups

Forty-eight male specific pathogen-free (SPF) grade Sprague-Dawley (SD) rats weighing 280–320 g were purchased from Beijing Vital River Laboratory Animal Technology Co. Ltd. (Beijing, China). The rats were housed under barrier-sustained conditions at 25°C with a 12 h light/dark cycle and *ad libitum* access to water and food for 5 d prior to the operation. The rats were randomly divided into two groups: A. normal intestinal lymph drainage (N+D), and B. I/R + intestinal lymph drainage (I/R+D) (n = 24 per group).

### Intestinal I/R and specimen collection

#### Intestinal I/R and lymph drainage

All surgical instruments, tubes for lymph collection (Nunc A/S, Roskilde, Denmark), artery clamps, and pipette tips were sterilized and confirmed pyrogen-free in advance. The rats were anesthetized with an intraperitoneal injection of 1% sodium pentobarbital (50 mg/kg). A midline incision was performed to separate the superior mesenteric artery (SMA) and intestinal lymphatic trunk. In the B group (I/R+D), the SMA was occluded for 60 min using an artery clamp, followed by reperfusion for 120 min. A small incision was made at the proximal end of the intestinal lymphatic trunk and a catheter (Jinan Medical Silicone Tube Plant, China) was inserted into the incision obliquely 3–5 mm towards the distal end. A small amount of medical adhesive (Beijing FuAiLe Science and Technology Development Co. Ltd., Beijing, China) was used on the serosa adjacent to the right kidney to fix the catheter. Outflow of lymph from the catheter was collected in a sterile test-tube for 180 min. The rats in the A group (N+D) were drained of lymph fluid for 180 min without clamping the SMA. After the operation, the collected lymph fluid (0.6–1.2 ml per rat) was centrifuged at 4°C for 15 min at 13,800g, and the supernatant was stored in sterile tubes at −80°C.

### Cells and culture conditions

The monocyte-macrophage cell line J774A.1 was purchased from the Cell Resource Center of School of Basic Medicine Beijing Union Medical College (Beijing, China) and cultured in high-glucose Dulbecco’s Modified Eagle Medium (DMEM) containing 10% fetal bovine serum (FBS) and 100 μg/ml penicillin and streptomycin. The cells were cultured to a logarithmic growth phase at 37°C in a humidified incubator with an atmosphere of 5% CO_2_. The cells were stimulated with lymph from each sub-group for 24 hours. Stimulation media consisted of the following preparations with a final concentration of 5% lymph fluid. A. Normal intestinal lymph drainage (N+D); and B. I/R + intestinal lymph drainage (I/R+D). Each group was divided into four sub-groups (n = 8), with different treatments as follows:

1. A1, B1 (Ly, I/R Ly): Lymph fluid added to normal medium without treatment.
2. A2, B2 (Ly PD, I/R PD): After adding proteinase K (20 mg/ml) into lymph fluid (5:2 v/v), the lymph was incubated at 55°C for 40 min to degrade the protein. The treated lymph as then added to the normal growth medium.
3. A3, B3 (Ly ER, I/R ER): Endotoxin removal columns containing immobilized polymyxin B that binds and removes endotoxin (Detoxi-Gel Endotoxin Removing Columns, Pierce, Biotechnology, Rockford, IL, USA) were used according to the manufacturer’s instructions. After treatment, the treated lymph was added to the normal medium.
4. A4, B4 (Ly PD+ER, I/R PD+ER): The lymph fluid was treated by both deproteinization and endotoxin removal prior to being added to the normal medium.

### Sample evaluation

#### Determination of protein content of the lymph fluid

A Coomassie brilliant blue protein measurement kit (Jiancheng Institute of Biology and Engineering, Nanjing, China) was used. The levels of protein in the drained lymph fluid were measured both before and after proteolysis at an absorption wave length of 595 nm (UV-Vis8550, double beam ultraviolet light/visible light absorption apparatus, Tianmei Science Technology Co., Ltd, Shanghai, China).

#### Determination of endotoxin levels in the intestinal lymph

A chromogenic limulus assay kit (Yi Hua Medical Technology Co., Ltd., Shanghai, China) was used at an absorption wave length of 545 nm for quantitative detection of lymph endotoxin both before and after treatment with polymyxin B agarose columns.

#### Enzyme-linked immunosorbent assays (ELISAs)

Tumor necrosis factor α (TNF-α), interleukin 1β (IL-1β), IL-6, soluble cell adhesion molecule (sICAM-1), macrophage chemoattractant protein-1 (MCP-1), macrophage inflammatory protein-2 (MIP-2), TLR4 and HMGB1 concentration from the lymph fluid, the monocyte-macrophage cell line and the supernatant of the stimulated cell line were determined using ELISA kits (Sun Biomedical Technology Co., Ltd., Beijing, China) according to the manufacturer’s protocols.

#### Western blot analysis of TLR4, NF-κBp65 and HMGB1 expression

Total protein extracts was prepared and samples were separated using SDS polyacrylamide gels. Proteins were then transferred to nitrocellulose membranes overnight at 4°C and blocked for 8 h with 5% bovine-specific albumin (BSA). The membranes were then incubated overnight with anti-TLR4, NF-κBp65 and HMGB1 primary antibody (1 μg/ml, ABCAM Ltd, Cambridge, UK) diluted in blocking solution (1:500, Beijing Biosynthesis Biotechnology Co., Ltd., China). Membranes were washed in Tris-buffered saline containing Tween (0.05%,TBST) and incubated with horseradish peroxidase-conjugated mouse secondary antibodies in 5% milk (1:3000, Santa Cruz Biotechnology Inc., Dallas, TX, USA) for 1 h at room temperature. Protein bands were visualized using an enhanced chemiluminescence (ECL) kit.

#### Quantitative real-time-PCR analysis of the gene expression of TLR4, HMGB1, NF-κBp65, MCP-1 and MIP-2

Total RNA was isolated from the stimulated cells with Trizol reagent (Invitrogen Corporation, Life Technologies, Carlsbad, CA, USA), followed by DNase digestion and repurification according to the manufacturer’s instructions. cDNA was prepared from 5 μg of RNA using a Reverse Transcription System (Applied Biosystems, Foster City, CA, USA) and was subjected to quantitative real-time PCR analysis with a TLR4 gene-specific primer (5′-TGCACACATCATTTGCTCAGCT-3′). Real-time PCR data were analyzed using the 2^-△△CT^ method as previously described [7].

### Statistical analysis

Quantitative data are presented as means ± standard deviation (SD). SPSS version 17.0 statistical software (SPSS, Inc., Chicago, IL, USA) was used to test the homogeneity of variance. The comparison between A1 and B1 groups was performed with a Student’s t test. Multiple comparisons were performed with one-way analysis of variance (ANOVA) followed by the least-significant difference (LSD) test. Statistical significance was defined as a *P*-value of *P* < 0.05.

## Results

### Levels of active factors in the lymph fluid after ischemia reperfusion

**Fig 1:**
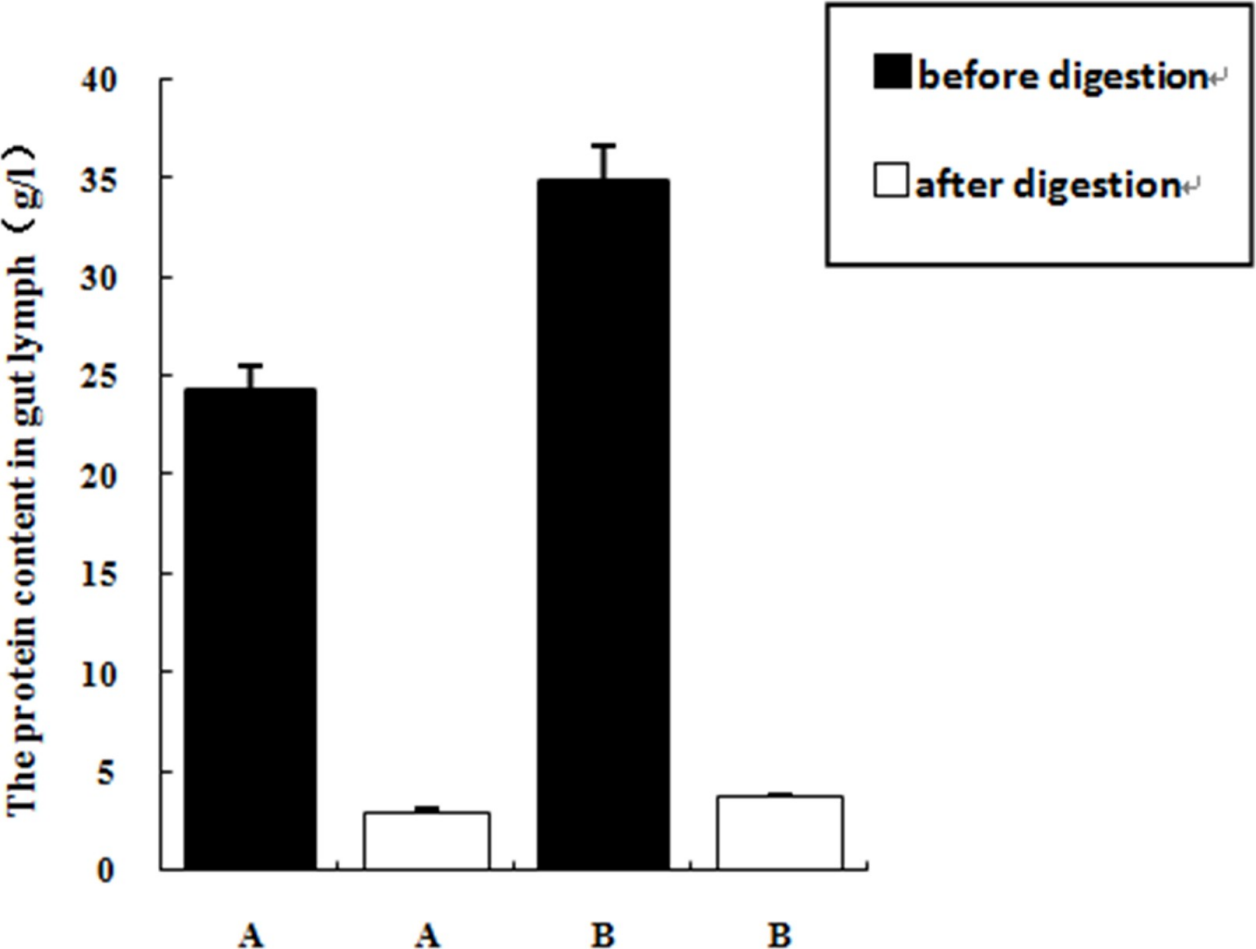
The protein content before and after the lysing of proteinase K (n = 24, X±SD). Note: ①. A: Normal intestinal lymph drainage (N+D); ②. B: I/R +intestinal lymph drainage (I/R+D).

**Fig 2:**
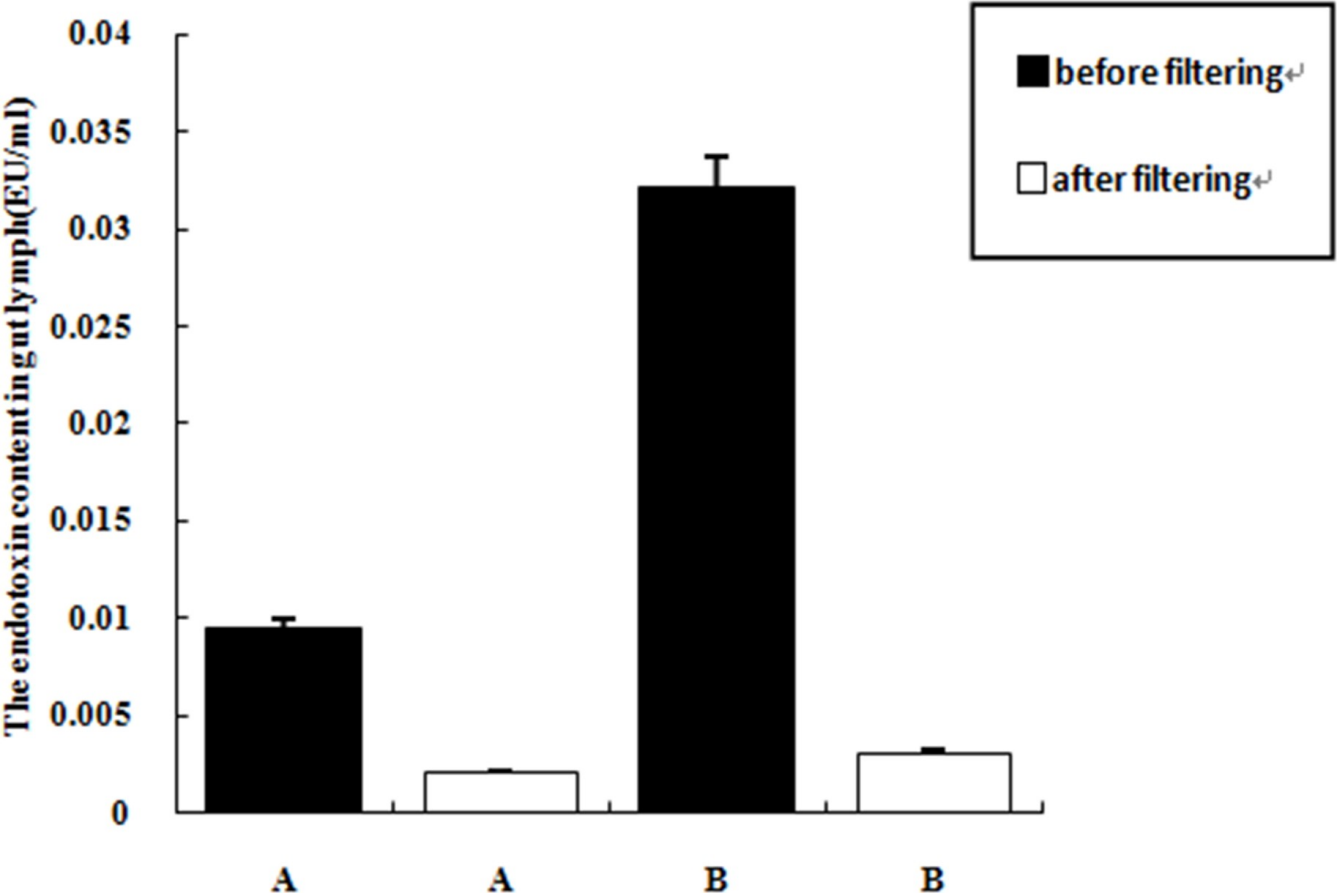
The levels of endotoxin in both before and after the treatment of endotoxin removing columns. Note: ①. A: Normal intestinal lymph drainage (N+D); ②. B: I/R +intestinal lymph drainage (I/R+D).

The levels of total protein, endotoxin, MCP-1, MIP-2, TLR4 and HMGB1 in the lymph fluid in group B (I/R+D) were significantly higher than those of group A (N+D) (*P* < 0.05), indicating that intestinal I/R increased the level of proinflammatory factors in the gut lymph fluid. Proteinase K treatment degraded most of the protein present in the intestinal lymph fluid. The efficiency of proteolysis in group A2 and group B2 were 87.91% and 89.32%, respectively. The endotoxin removal columns removed the majority of the endotoxin present in the gut lymph fluid; with efficiencies of 78.40% in group A3, and 90.61% in group B3. (Table 1; Figs 1 and 2)

**Table 1:** The protein content in the lymph fluid in two groups (n = 24, X±SD).

Groups	Protein (g/l)	Endotoxin (EU/ml)	MCP-1(pg/ml)	MIP-2(ng/ml)	TLR4(ng/ml)	HMGB1(ng/ml)
A B	24.35±1.37 34.89±1.68^a^	0.0095±0.0053 0.0321±0.0131^a^	49.71±6.72 87.48±11.57^a^	1.25±0.53 2.40±0.77^a^	0.29±0.054 0.44±0.061^a^	5.24±0.48 6.26±0.53^a^

①. A: Normal intestinal lymph drainage (N+D); ②. B: I/R+intestinal lymph drainage (I/R+D)

a: compare with A group, P<0.05

### Expression of cytokines and chemokines by macrophages stimulated with lymph fluid *in vitro*

The expression of TNF-α, IL-1β, IL-6, sICAM-1, MCP-1, MIP-2 and HMGB1 by macrophages in group B1 were significantly higher than those of group A1 (*P* < 0.05), both in the cell cytoplasm and in the supernatant. Pretreatment of the lymph by protein degradation (groups A2 and B2) and protein degradation + endotoxin removal treatment (groups A4 and B4) significantly decreased the above factors compared with the untreated group (A1 and B1) (*P* < 0.05). However, endotoxin removal did not reduce the expression of these proinflammatory factors, with no significant difference between groups A1 and A3, and groups B1 and B3. This suggests that a protein component of the lymph fluid induced by I/R, that was not endotoxin, increased the expression of proinflammatory factors, chemotactic factors and HMGB1 by macrophages *in vitro*. (Tables 2 and 3)

**Table 2:** The concentrations of inflammatory factors in cells (pg/ml).

Groups	TNF-α	IL-1β	IL-6	sICAM-1
A1	42.99±1.61	34.54±1.41	38.11±1.41	0.57±0.02
A2	38.79±1.83^a^	30.79±1.09^a^	35.25±1.15^a^	0.49±0.31^a^
A3	42.01±1.38^d^	33.96±1.46^d^	37.47±1.56^d^	0.56±0.16^d^
A4	37.88±1.34^a^	29.39±2.14^a^	35.41±1.66^a^	0.49±0.07^a^
B1	49.46±1.42^a^	38.05±1.33^a^	43.38±1.29^a^	0.61±0.05^a^
B2	41.05±2.03^b^	33.87±1.09^b^	39.82±1.14^b^	0.54±0.12^b^
B3	48.13±1.63^e^	37.45±1.63^e^	42.45±1.51^e^	0.59±0.08^e^
B4	40.63±1.44^b^	33.32±2.05^b^	39.59±1.61^b^	0.53±0.02^b^

①. A: Normal intestinal lymph drainage (N+D); ②. B: I/R+intestinal lymph drainage (I/R+D)

a: compare with A1 group, P<0.05

b: compare with B1 group, P<0.01

d: compare with A4 group, P<0.01

e: compared with B4 group, P<0.01

**Table 3:** The concentrations of inflammatory factors in the supernatant of the cells (pg/ml).

Groups	TNF-α	IL-1β	IL-6	sICAM-1	MCP-1	MIP-2	HMGB1
A1	180.06±1.73	121.35±1.65	165.67±2.03	4.38±0.04	162.15±2.19	166.94±2.00	945.64±5.07
A2	165.95±2.19^a^	103.87±1.64^a^	160.33±2.25^a^	4.09±0.06 ^a^	152.15±1.56^a^	162.82±2.45^a^	939.24±4.77^a^^,^^d^
A3	179.21±1.55^d^	120.32±1.70^d^	165.17±2.63^d^	4.30±0.06^d^	160.81±2.55^d^	165.99±1.50^d^	943.29±4.47^d^
A4	165.09±1.23^a^	103.01±1.82^a^	160.78±2.09^a^	4.03±0.14^a^	152.65±1.61^a^	161.02±2.033^a^	938.85±5.42^a^
B1	203.42±2.08^a^	136.49±1.66^a^	186.97±1.794^a^	4.99±0.03^a^	182.93±1.611^a^	190.17±1.27^a^	1018.49±5.21^a^
B2	192.95±1.86^b^	123.44±1.86^b^	170.97±2.55^b^	4.78±0.04^b^	175.79±2.13^b^	185.88±1.48^b^	993.64±5.59^b^
B3	202.51±1.56^e^	135.63±1.36^e^	186.21±2.63^b^^,^^e^	4.80±0.03^e^	180.74±1.67^e^	189.59±1.72^e^	1016.37±6.39^e^
B4	192.42±1.21^b^	124.24±2.01^b^	170.41±1.61^b^	4.76±0.05^b^	174.27±1.56^b^	185.27±2.75^b^	993.74±5.19^b^

①. A1, B1 (Ly, I/R Ly);②. A2, B2 (Ly PD, I/R PD);③. A3, B3 (Ly ER, I/R ER);④. A4, B4 (Ly PD+ER, I/R PD+ER)

a: compare with A1 group, P<0.05

b: compare with B1 group, P<0.01

d: compare with A4 group, P<0.01

e: compared with B4 group, P<0.01

### Upregulation of signaling molecules by macrophages after stimulation with lymph fluid

**Fig 3:**
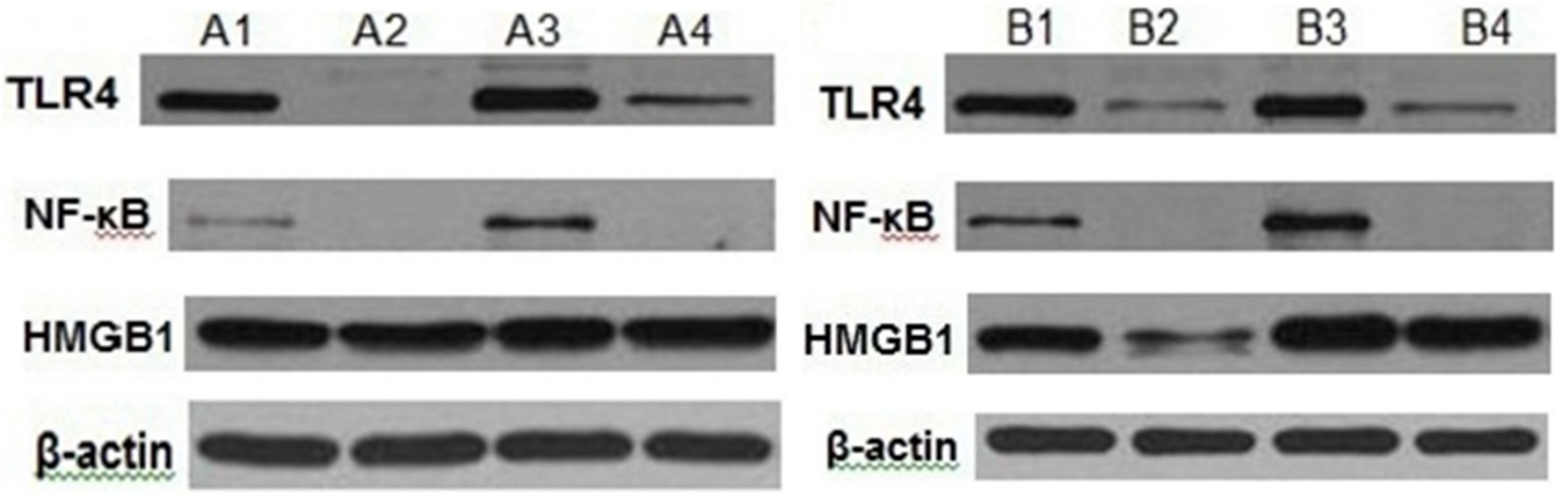
The protein expression of each group. Note: ①. A1, B1 (Ly, I/R Ly); ②. A2, B2 (Ly PD, I/R PD); ③. A3, B3 (Ly ER, I/R ER); ④. A4, B4 (Ly PD+ER, I/R PD+ER).

The protein expression of TLR4 and NF-κBp65 in group B1 was significantly higher than group A1. However, the protein expression of TLR4 and NF-κBp65 of groups B2 and B4 were significantly decreased compared with group B1. The same trend was seen in group A. There were no significant changes between B1and B3, A1 and A3 in TLR4, NF-κBp65 and HMGB1. Data indicated that the lymph fluid after intestinal I/R elevated the protein expression of TLR4 and NF-κBp65 in macrophages *in vitro*. Removing the protein in lymph fluid significantly attenuated the expression of TLR4 and NF-κBp65. Endotoxin removal did not significantly lower the expression of TLR4, NF-κBp65 and HMGB1 (Fig 3).

### Gene expressions in macrophage *in vitro* after stimulation with lymph fluid

The levels of TLR4, HMGB1, NF-κBp65, MCP-1 and MIP-2 mRNA of group B1 were significantly higher than those of group A1 (*P* < 0.01). By comparing A2 and A4 with A1, and B2 and B4 with B1, we found that these factors were significantly decreased in A2 and A4, and B2 and B4, respectively (*P* < 0.01). Protein degradation (groups A2 and B2) and protein degradation + endotoxin removing treatment (groups A4 and B4) significantly decreased these factors. Comparing A3 with A1, B3 with B1, the expressions of these factors were not significantly decreased in A3 and B3 respectively. This indicates that proinflammatory proteins, but not endotoxin, present in the lymph fluid after intestinal I/R can increase the expression of TLR4, NF-κBp65, MCP-1 and MIP-2 *in vitro*. (Table 4)

**Table 4:** The gene expression in each group (N = 2^-△△Ct^).

Groups	TLR4	HMGB1	NF-κBp65	MCP-1	MIP-2
A1	2.12±0.45	1.92±0.47	2.63±0.32	87.77±2.20	26.66±1.27
A2	1.76±0.23^a^	1.42±0.40^a^	1.97±0.17^a^	73.21±0.17^a^	21.86±0.85^a^
A3	2.11±1.98^d^	1.92±0.42^d^	2.56±0.31^d^	86.32±0.52^d^	25.97±3.42^d^
A4	1.74±0.30^a^	1.41±0.31^a^	1.91±0.29^a^	73.61±0.11^a^	20.37±0.43^a^
B1	2.86±0.24^a^	2.22±0.27^a^	3.93±0.12^a^	94.84±1.19^a^	40.51±0.72^a^
B2	1.76±0.79^b^	1.60±0.13	1.55±0.26^b^	80.22±0.07^b^	35.06±2.62^b^
B3	2.80±0.62^e^	2.11±0.50	3.89±0.05^e^	93.27±0.40^e^	40.51±3.71^e^
B4	1.76±0.46^b^	2.09±0.22	1.57±0.29^b^	80.08±0.07^b^	30.49±1.31^b^

①. A1, B1 (Ly, I/R Ly);②. A2, B2 (Ly PD, I/R PD);③. A3, B3 (Ly ER, I/R ER);④. A4, B4 (Ly PD+ER, I/R PD+ER)

a: compare with A1 group, P<0.01

b: compare with B1 group, P<0.01

d: compare with A4 group, P<0.01

e: compared with B4 group, P<0.01

## Discussion

There are multiple steps to distant organ damage being caused by intestinal injury. First, the visceral vessel contracts as the intestines are under ischemia. Second, resuscitation causes reperfusion injury. Third, there is an interaction between trypsin and intestinal ischemia [8]. Fourth, bacterial translocation [9] and their metabolic products enter the gut [10], which accelerates the activation of detrimental bio-active materials in the lymph fluid. In the state of injury, caused by ischemia, shock and major surgery, it could trigger serious inflammatory reactions. All these are the foundation for the intestine being the engine of multiple organ dysfunction syndromes (MODS), although the exact mechanism of pathogenesis is still under debate [11, 12]. The intestine causes a cascade of activation of proinflammatory factors and cytokines, causing the bacterial flora to change, leading to an overgrowth of bacteria and intestinal barrier damage. The further spread of inflammation may be mediated by the “gut-lymph” pathway, which is how bacteria in the gut lymph after intestinal damage and bio-active materials can enter the thoracic duct, through the intestinal lymphatic system, and then spread throughout the whole body. Additionally, whole-body infection and/or MODS could take place under the circumstances where there is no bacterial dislocation in the portal or body circulations.

As the gut is the engine of MODS, what is the exact pathway(s) involved in the pathogenesis of MODS, and how they are involved? The lymph fluid from trauma-hemorrhagic shock (T/HS) animals transfused into healthy animals can cause lung injury [12]. Researchers infer that in hemorrhagic shock, bacteria and associated toxins may pass through the lymph pathway to the lung and subsequently cause lung injury [13–15]. Research indicates that lymph fluid is the key factor linking the trauma hemorrhagic shock (T/HS) and MODS. In the progression of intestine-caused MODS, apart from bacteria and the effect of toxins, some proinflammatory and bioactive factors produced by gut injury may participate in the process. Feinman et al [16] found that in the T/HS animal model, hypoxia-inducible factor-1α (HIF-1α) deficient mice exhibited the amelioration of I/R-induced intestinal barrier damage and bacteria dislocation. In our experiment, the lymph fluid of group B had significant higher levels of endotoxin, TLR4 and HMGB1 compared with group A, suggesting that during intestinal I/R, endotoxin, TLR4 and HMGB1 were likely to enter the systemic circulation via the “gut-lymph” pathway. After intestinal I/R, general inflammatory injury in the intestinal mucosa is correlated with a strong innate immune response, mediated by activation of the TLR-NF-κB-cytokine pathway [17]. In a recent study by Ben et al [18], TLR4 deficient mice exhibited a significantly lower expression of TNF-α, IL-6, MCP-1 and MIP-2 after gut ischemia-reperfusion compared with TLR4 wild type mice. The chemokine super-family comprises many small proteins of 6–8 kD, and their structures are highly homologous. The characteristic motif is the cysteine residue in the sequence. According to the number and arrangement of cysteine residues, chemokines have three sub-families: α, β and γ. The α sub-family, also called CXC sub-family, are mostly neutrophil associated chemokines, and their sequence is a cys-x-cys motif. The β sub-family includes MIP-2 and IL-8. In the current report, the MCP-1 and MIP-2 levels in the lymph fluid of group B were significantly higher than those of group A (*P* < 0.05), indicating that after I/R, the intestine may produce or release these chemokines, which affect neutrophils and regulate the network of cytokines (the interaction between cytokine and cytokine, between cytokine and chemokine, respectively) to influence the chemotaxis of neutrophils.

The mesenteric lymph fluid has a crucial role in the pathogenesis of distant organ injury in critically ill patients [19]. Adams et al [20] demonstrated that the cause of endothelial cell death or tight junction loss between cells in trauma hemorrhagic shock was a factor present in the lymph fluid. They utilized the solid phase extraction and ion exchange chromatography to isolate large amount of abnormal protein fragments from rat lymph fluid after shock, and through gel electrophoresis and mass spectrometry they found that the bioactive materials were serum albumin and lipoproteins with altered structures. We used proteinase K to degrade all native proteins in the lymph fluid, such as HMGB1 and TLR4. The rates of efficiency of proteolysis in groups A and B were 78.4% and 90.61% respectively. Our methods may lend credit to further research for analyzing the content of lymph protein.

Endotoxin, a type of lipopolysaccharide, is one part of Gram negative bacterial cell wall. In this experiment, the endotoxin level of group B was significantly higher than that of group A (*P* < 0.05). It has been demonstrated that in intestinal I/R injury, endotoxin possibly through “gut-lymph” pathway to damage gut barrier to circulation system. We used high affinity endotoxin removal columns, which bind the lipid A structure of endotoxin to fixed polymyxin B, to remove the endotoxin. With the very low concentration of endotoxin in group A: 0.0095 ± 0.0053 EU/ml, we only achieved an efficiency of 78.40%. However, in group B, the efficiency reached 90.61%, suggesting that the methods of using endotoxin columns had some merit. In group B, the expression of TLR4 and NF-κBp65 in group B2 and B4 was significantly lower than those of B1. But there was no significant difference between B3 and B1 in TLR4 and NF-κB. The degradation of protein in lymph fluid significantly decreased the level of TNF-α, IL-1β, IL-6, sICAM-1 and the expression of TLR4 and NF-κBp65 by macrophages. Endotoxin removal did not significantly decrease the level of TLR4, NF-κBp65, inflammatory factors and chemotactic factors. For the low concentration of endotoxin, the effects of endotoxin may not enough to trigger this reaction. Reino et al [21] observed that lymph samples did not contain detectable levels of bacteria, endotoxin or bacterial DNA. This may have been related to the minimum detection limit of 0.06 EU/ml.

## Conclusions

In our study, the lymph fluid after intestinal I/R contains increased levels of proinflammatory mediators. Deproteinization of the lymph fluid significantly decreased the concentration of proinflammatory factors, chemotactic factors, TLR4 and NF-κBp65 in cell cultures stimulated with the lymph fluid. However, removal of endotoxin from lymph fluid did not significantly decrease the expression of bioactive factors, suggesting that it may be the proteins, not the endotoxin, present in gut lymph after intestinal I/R that triggers the inflammatory reaction *in vitro*. However, besides TLR4, are there any other mediators in the lymph fluid that can cause organ injury in intestinal I/R? This question will be further researched in continuing studies.
